# Healthcare providers perspectives on opportunities and challenges for adopting comprehensive primary healthcare-seeking behaviour in selected districts in Tanzania

**DOI:** 10.4102/jphia.v17i1.1573

**Published:** 2026-05-05

**Authors:** Alphoncina Kagaigai, Pankras Luoga, Novatus Tesha, Malale Tungu, Godfrey Swai, Mangi Ezekiel, Nathanael Sirili, Amani Anaeli

**Affiliations:** 1 Department of Development Studies, School of Public Health and Social Sciences, Muhimbili University of Health and Allied Sciences, Dar es Salaam, United Republic of Tanzania; 2 Private Public Health Consultant, Dar es Salaam, United Republic of Tanzania; 3 Department of Behavioural Sciences, School of Public Health and Social Sciences, Muhimbili University of Health and Allied Sciences, Dar es Salaam, United Republic of Tanzania

**Keywords:** health-seeking behaviour, primary health care, universal health coverage, Tanzania, qualitative study

## Abstract

**Background:**

Comprehensive primary healthcare (PHC) can address over 80% of global health needs across a lifetime. Adopting health-seeking behaviour (HSB) within PHC frameworks is critical for achieving Universal Health Coverage (UHC) and Resilient and Sustainable Systems for Health (RSSH) by 2030.

**Aim:**

Evidence on the adaptation of comprehensive PHC-HSB in Tanzania remains limited. This study explored opportunities and challenges for adopting comprehensive primary healthcare-seeking behaviour (PHC-HSB) in two districts of Tanzania.

**Setting:**

This study was conducted in two district councils (DCs) in Tanzania (Iringa and Sumbawanga DCs) selected based on the population served by skilled healthcare workers (HCWs).

**Methods:**

An exploratory qualitative case study design was employed, using in-depth interviews (IDIs) with HCWs and community health workers (CHWs). Data were analysed thematically using NVivo software.

**Results:**

The study found that key opportunities for adopting comprehensive HSB include the existence of exemptions and waiver, presence of special clinics, benefits of early seeking behaviour, availability of outreach services, community’s interest in health issues, presence of CHWs and health education provided at the household level.

**Conclusion:**

The challenges mentioned were frequent stockout of drugs, overburdened health facilities (HFs) because of large catchment areas, cultural and behavioural barriers to timely health-seeking, unreliable and inadequate transport systems, poor continuity of preventive health-seeking after campaigns, and low health insurance coverage.

**Contribution:**

Adopting comprehensive HSB in Tanzania is feasible but requires government efforts to strengthen infrastructure, drug supply chains and community education.

## Introduction

Primary healthcare (PHC) remains the cornerstone of equitable and sustainable health systems worldwide, with the potential to address over 80% of health needs across an individual’s lifetime.^1^ Since the Alma-Ata Declaration of 1978, PHC has been recognised as a critical strategy for achieving Universal Health Coverage (UHC) and reducing health disparities, particularly in low- and middle-income countries (LMICs).^2^ Tanzania, like many sub-Saharan African nations, has formally adopted PHC principles, integrating them into national policies, such as the Tanzania Essential Health Package of Interventions (TEHPI).^3^ However, despite decades of PHC implementation, healthcare access remains fragmented, with a significant proportion of the population still relying on higher-level facilities, informal care providers or delaying treatment because of structural and socio-cultural barriers.^1,4^

The Astana Declaration (2018)^5^ reaffirmed the importance of comprehensive primary healthcare, emphasising the need for a shift from passive treatment-seeking behaviour (TSB), where individuals seek care only when ill, to proactive health-seeking behaviour and practices (HSB), which include preventive and early detection measures.^6^ This transformation is particularly crucial in Tanzania, where only 38% of healthcare consultations occur at primary-level facilities (dispensaries, health centres and district hospitals), while 62% are conducted at higher-level hospitals, pharmacies or specialised facilities.^7^ Such reliance on reactive care contributes to late disease presentation, increased healthcare costs and preventable morbidity and mortality, particularly for chronic conditions like hypertension and diabetes.^8^

The comprehensive primary healthcare framework outlined in the Astana Declaration can potentially expand healthcare coverage in Tanzania from 50% to 90%, bringing the nation closer to Universal Health Coverage targets.^8^ Tanzania’s health system operates under a pyramidal referral structure, with first-level of PHC comprising dispensaries (*n* = 8446), health centres (*n* = 1282), and primary referral district hospitals (*n* = 183). The second level is the tertiary facilities comprised of regional referral hospitals (RRH) (28), zonal referral hospitals (ZRH) (5) and to the highly equipped specialised (6) and national hospitals (https://hfrs.moh.go.tz/web/index.php).^9^ However, inequitable resource allocation undermines primary healthcare efficiency persists, with only 14.3% of health expenditure directed toward primary care, while the majority of the funds (85.7%) is allocated to higher-level services.^10^ This imbalance contributes to Tanzania’s low UHC Service Coverage Index (*n* = 43/100) and leaves 3.8% of households vulnerable to catastrophic health expenditures.^11,12^

Similarly, Tanzania aligns with the global agenda to end pandemics and attain Universal Health Coverage (UHC) by 2030. Tanzania is therefore obliged to fundamentally transform from the current TEHPI with a focus on TSB towards the futuristic comprehensive primary healthcare framework of health-seeking behaviour. While the UHC Service Coverage Index (SDG 3.1.8) improved from 37 in 2010 to 46 in 2019 for Tanzania, the proportion of households spending over 10% of their income on health has increased fivefold from 3.79% in 2011 to 15% in 2023.^13,14^ The devastating impact of the COVID-19 pandemic negatively impacted the fragile primary healthcare framework, which is operating on costly treatment-seeking behaviour, thereby hindering progress towards strengthening pandemic preparedness and the attainment of Universal Health Coverage by 2030.^15^

Previous studies had limited coverage and mostly focused on prevalence, drivers and determinants of health-seeking behaviours of sub-populations, including mothers and caregivers of under-5-year-old children for specific diseases in Tanzania,^16,17,18^ There is limited empirical evidence on the available opportunities and challenges in the adaptability of comprehensive primary healthcare-seeking behaviour (PHC-HSB) at PHC settings at district councils (DCs) in Tanzania. This study, therefore, addresses this gap by exploring opportunities for adopting comprehensive PHC-HSB in selected Tanzanian districts as well as identifying systemic and cultural challenges hindering proactive health-seeking. The findings inform policy recommendations to strengthen PHC delivery and accelerate progress toward UHC goals.

## Research methods and design

### Study design

An exploratory qualitative case study design was conducted in Iringa and Sumbawanga DCs in Tanzania. A purposive sampling technique was used to select the study districts’ DCs based on the number of the population served by trained healthcare providers in the study settings (Table 1). Iringa DC was selected for having the highest number of people served by the trained healthcare providers, while Sumbawanga DC was chosen for having the lowest number. In-depth interviews (IDIs) were conducted among healthcare workers (HCWs) to get a comprehensive understanding of the challenges and opportunities for adopting a comprehensive PHC-HSBs in the selected two DCs. The case study design was deemed appropriate to study a comprehensive PHC-HSB, which is a complex and non-linear phenomenon involving social processes.^19^

**TABLE 1:** Background information of the study participants.

Serial number	Sex	Cadre	Facility type	District council
1	M	Assistant nurse	Dispensary	Sumbawanga
2	M	CHW	Dispensary	Sumbawanga
3	F	Nurse in charge	Dispensary	Sumbawanga
4	M	CHW	Health centre	Sumbawanga
5	F	Nurse	Health centre	Sumbawanga
6	M	CHW	Dispensary	Sumbawanga
7	M	Clinical officer	Dispensary	Sumbawanga
8	F	Nurse	Dispensary	Sumbawanga
9	F	Medical officer in charge (MD)	Hospital	Sumbawanga
10	M	CHW	Health centre	Iringa
11	M	Clinical officer	Health centre	Iringa
12	M	Medical officer in charge	Health centre	Iringa
13	M	CHW	Dispensary	Iringa
14	F	Medical officer in charge (MD)	Hospital	Iringa
15	M	CHW	Hospital	Iringa

M, male; F, female; CHW, community health worker; MD, medical doctor.

### Study setting

The study was conducted in two councils (Iringa and Sumbawanga DCs) from two regions out of 26 regions in Tanzania. Iringa DC is among the five councils of the Iringa Region. This council is situated in the Southern highland of Tanzania between latitudes 6.540 and 100 and longitudes 33 and 370–000. Administratively, the council is divided into six divisions and 25 wards with a total of 123 villages and 718 hamlets distributed unevenly. According to the 2022 Census, the district had a population of 315 354 of which 153 556 are males and 161 798 females. The economic activities are agriculture, fishing and livestock keeping in this district. Additionally, the district has about 77 health facilities (HFs) (8 health centres, 68 dispensaries). Tanzania has a hierarchical health system with the dispensaries at the bottom, found in every village, followed by health centres found at the ward level. District hospitals are found at the district level, and at the regional level we have regional referral hospitals. The tertiary level is comprised with the zone hospitals, and at a national level, there is a national hospital, and 1 district hospital served by 318 skilled HCWs.

Sumbawanga DC is one of the three councils of Rukwa region situated in the South-west highlands of Tanzania on latitudes 7.8 and 9 South, longitudes 31 and 32.3 to the East. Administratively, the council has seven (*n* = 7) divisions, 23 wards, and 167 villages. According to the 2022 Census, the district had a population of 303 986 of which 144 657 are males and 159 329 females. The economic activities are agriculture, livestock keeping, tourism, beekeeping and fishing. The total number of HFs available is 76 comprising two (*n* = 2) hospitals, eight (*n* = 8) health centres and 66 dispensaries. These facilities are served by 260 skilled HCWs.

### Study participants and sampling strategies

The study population included community health workers (CHWs) and the HCWs at the selected facilities (dispensaries, health centres and district hospitals). Overall, the study included 15 participants: six CHWs and nine HCWs from the sampled primary HFs. Data were collected from two DCs of Iringa and Sumbawanga. The study was designed as a PHC facility-based since most of the information was obtained from healthcare providers at the facility. A multistage sampling strategy was employed during the sampling process. In the first stage, a purposive sampling technique was applied to select two regions among 26 regions. In the second stage, two DCs were purposively selected based on the number of people served by trained healthcare providers and by considering the rural characteristics of councils. In the third stage, from each DC, we purposively selected a DC hospital, health centres and dispensaries as primary HFs. At the fourth stage, purposive sampling was used to select individual participants for IDIs. Healthcare workers were eligible if they were facility in-charges or frontline HCWs (e.g. clinicians or nurses) with at least 1 year of experience at the selected facility. Community health workers were selected based on their active role in community-level health activities linked to the facility and their experience supporting service delivery in the facility’s catchment area. Efforts were made to include participants from different facility levels (dispensary, health centres and district hospital) and cadres to capture diverse perspectives on HSB within PHC settings. In total, 15 participants (6 CHWs and 9 HCWs) were interviewed across the selected facilities.

### Data collection techniques and tools

The study employed IDIs qualitative data collection methods to explore challenges and opportunities in adopting comprehensive PHC-HSB. Participants included CHWs and trained HCWs from selected HFs in Iringa and Sumbawanga districts. Interviews were conducted in a facility-based setting, focusing on individual and community health-seeking behaviours. The qualitative approach allowed for an in-depth understanding of the complex and non-linear social processes influencing PHC-HSB.

The interview guide consisted of questions on participants’ views and perceptions regarding HSB in their area of work, as well as opportunities and challenges with adopting health-seeking behaviour. We interviewed one to two HCWs from each selected HF. In each DC, interviews were conducted with seven HCWs who were purposely selected. Each interview was conducted by two researchers, where one facilitated the interview and another one facilitated note taking, audio recording, and probing. All interviews were conducted in Swahili language, which is widely understood in Tanzania. All interviews were audio-recorded using a digital voice recorder, and the duration of the interview ranged from 30 min to 45 min. Debriefings were conducted after every interview to ensure consistency, quality control, and to capture new emerging issues.

To ensure trustworthiness of the findings, we included participants from different cadres (HCWs and CHWs), which supported credibility through triangulation of perspectives. Transferability was strengthened by providing a detailed description of the study setting. Dependability was enhanced by documenting the data collection procedures and analysis process in detail, thereby supporting the reliability of the findings.

### Data analysis

Data analysis began with reading and re-reading the interview transcripts to achieve familiarity with the data. The transcripts were then systematically coded by identifying meaningful units and extracting key statements related to health-seeking behaviours. Codes were compared and grouped into categories, which were subsequently organised into sub-themes and overarching themes based on patterns across participants’ responses. The analysis followed an inductive thematic approach, allowing themes to emerge from the data rather than being predetermined. NVivo 14 software was used to support coding and data management. The case study design facilitated an in-depth exploration of contextual factors influencing health-seeking behaviours, thereby providing a comprehensive understanding of the phenomenon. Thematic saturation was considered achieved when no new codes or themes emerged from successive interviews, hence strengthening the robustness of the findings.

### Ethical considerations

Ethical clearance to conduct this study was obtained from the Muhimbili University of Health and Allied Sciences Ethical Review Board with ethical clearance number: MUHAS-REC-04-2022-1121. Permission to conduct the study in two districts (Iringa and Sumbawanga DCs) was requested from the Regional Administrative Secretary (RAS) of the respective region, then District Administrative Secretaries (DAS) and District Medical Officer (DMOs) of the two districts after submitting an introduction letter and the ethical approval from Muhimbili University of Health and Allied Sciences (MUHAS).

## Results

### Participants’ background information

The study comprised 15 participants, of which 10 were male and 5 were female. The participants had a mixture of different cadres including nurses, clinical officers, medical doctors and CHWs. Out of the 10 sampled facilities, two were district hospitals, three were health centres and five were dispensaries. The respondents come a small group of healthcare workers who can easily be identified, thus, no additional participant identifiers are provided.

### Results summary

The summary of the study findings is presented in Table 2. The findings are structured into two themes, namely, evidence of opportunities (enablers) for adopting comprehensive PHC-HSBs and evidence of challenges (barriers) for adopting PHC-HSBs.

**TABLE 2:** A summary table of developed codes, sub-themes and themes.

Themes	Sub-themes	Codes
1. Evidence of Opportunities (Enablers) for adopting Comprehensive HSB	1.1. Existence of exemptions and waiver	Presence, existence, not paying, exemptions, waiver, free of charge, not charged for the services
	1.2. Presence of special clinics	Special clinic, have own services, special arrangements, high blood pressure, diabetes
	1.3. Benefits of early seeking behaviour	Come before they are sick, benefits of early seeking, get education, get time for longer discussions with health providers, get to know health condition, assist in assessing changes
	1.4. Availability of outreach services	Outgoing health services, outreach services, going to their places/houses, visiting their residence/homes, providing services near them
	1.5. Local community’s interest in hearing health issues	Community, support, local leaders, Support of community leaders, community’s interest
	1.6. Presence of community health workers	Community health worker, village health worker, our health worker, CHW
	1.7. Health education provided at the household level	Health education at home, household level
2. Evidences of Challenges for adopting Comprehensive HSB	2.1. Frequent stockout of drugs	No drugs, the challenge of medicine, unavailability of drugs/medicine, need to buy drugs, outside pharmacies, private pharmacies, drug stock out, drugs not available, shortage of medicine
	2.2. Overburdened health facilities because of large catchment areas	One dispensary, three villages, one health centre serving a number of villages, one health facility to many villages, Many and distant villages
	2.3. Cultural and behavioural barriers to timely health-seeking	Wait until sick, do not check regularly, if not sick.no habit to check, they come too late, come at late stage, Culture of health-seeking, People do not seek for health services if not sick, Culture (tendency) of local population, not seeking health services when they are not sick, some local beliefs
	2.4. Unreliable and inadequate transport systems	Bicycles and motor cycles, walking on foot, miss transport, limited challenge, Unreliable transport to reach the health facilities
	2.5. Poor continuity of preventive health-seeking after campaigns	Risks, behaviours, people attend only when there is a campaign/free service, no routine screening, personal challenges, people do not seek for health services if not sick
	2.6. Low health insurance coverage	Few use health insurance, only very few who are not covered by insurance afford the cost of health services

HSB, health-seeking behaviour; CHW, community health workers.

### Theme 1: Evidence of opportunities (enablers) for adopting comprehensive health-seeking behaviour

This theme was based on the evidence of the available opportunities in the PHC systems for adopting comprehensive health-seeking behaviour, which had seven sub-themes namely: existence of exemptions and waiver, presence of special clinics, benefits of early health-seeking behaviour, availability of outreach services, community’s interest in health issues, presence of CHWs and health education provided at the household level. These sub-themes are described as follows.

#### Sub-theme 1.1: The existence of exemptions and waivers for vulnerable groups

Participants reported that most of the HFs grant exemptions and waivers to vulnerable groups, including the elderly, pregnant women, under-five children and disabled people. These exemptions and waivers cover health services, including consultation fees, laboratory tests and drugs, which may increase the HSB among vulnerable groups. Since people who belong to these groups do not need to pay cash for health services, it helps them to be motivated to attend the HFs to seek health care services even if they do not have cash. One participant from Iringa DC mentioned that:

‘Exemption and waivers for accessing health services start with under-five children, disabled people, elderly and pregnant women. These groups access health services which are provided for free of charge. Services including laboratory tests, consultation fees, drugs and delivery services that might be difficult for them to pay.’ (Participant #6)

#### Sub-theme 1.2: Presence of special clinics for some chronic diseases

Participants explained that there are special clinics in some HFs that deal with some of the health conditions, like treatment for pneumonia, tuberculosis (TB), hypertension, diabetes and cervical cancer screening, which have special days to be offered. They indicated that having special clinics on certain days of the week makes more people seek healthcare services, as one participant from Sumbawanga DC commented:

‘Patients suffering from high blood pressure, diabetes and other chronic diseases have their clinics on Wednesday and Friday. These clinics help people with chronic diseases to increase health-seeking behaviour. Therefore, this may improve their health status.’ (Participant #1)

#### Sub-theme 1.3: The availability of outreach services

Most of the participants mentioned that there are some health services which are offered through outreach programmes in the catchment areas of that particular HF. This approach was described as a way of promoting comprehensive HSB by bringing services closer to people’s homes. One participant from Sumbawanga DC said:

‘We conduct outreach programmes to facilitate access to some important services closer to people who are living far from the health facility for motivating them to increase health-seeking behaviour. These services include health education, vaccination, medical tests like pressure, human immunodeficiency virus (HIV), and other related medical checkups for pregnant women and other vulnerable groups. Most medical checkups help people to know their health status early such as understanding their blood pressure, blood sugar, and body weight baseline. Therefore, there is a need for a special strategy to strengthen HSB among individuals to improve their health status.’ (Participant #2)

#### Sub-theme 1.4: Community’s interest in health issues

Participants reported that community members demonstrated strong interest in receiving health information and guidance from HCWs and CHWs, particularly on issues related to disease prevention, early identification of illness, and available health services. This interest was viewed as an important opportunity for promoting comprehensive health-seeking behaviour, as it created a conducive environment for health education, dialogue, and improved community engagement with preventive and promotive health services. In addition, participants emphasised that close relationships between health providers and community members facilitated trust and encouraged people to seek information and guidance when CHWs or HCWs visited their areas. Community leaders were also reported to provide full support for health-related initiatives, strengthening mobilisation and acceptance of community-based activities. As one participant from Iringa DC explained:

‘One of our achievements is the increased people’s attention to a healthy life and building close relationships with community members and HCWs including CHWs. When people see me in their areas, they become interested to know what I have come for them about health issues.’ (Participant #5)

#### Sub-theme 1.5: The presence of community health workers

Participants emphasised that the presence of CHWs within communities provides an important opportunity to adopt comprehensive health-seeking behaviour. CHWs were described as playing a critical role in providing health education at the household and at the community levels, raising awareness about disease prevention, early health-seeking, and available health services. Participants further explained that CHWs served as a vital link between communities and HFs, supporting referrals and follow-up and encouraging timely utilisation of services. As one HCW from Sumbawanga DC explained:

‘… There is something called PHC that incorporates CHWs which I think it is good. These CHWs are in the villages and know their people and their health challenges. It is crucial to build the capacity for these CHWs which is different from that of PHC. This is because CHWs act as the link between health facilities and the community. Wherever there is challenging question and fail to respond, they communicate with the respective health facility for help or go together to visit the family or villages with that challenge to intervene.’ (Participant #3)

#### Sub-theme 1.6: Health education provided at the household level

Participants explained that there is a high possibility of having comprehensive HSB since most of the CHWs visit most households to provide health education on various health topics. In addition, education is also provided at the HF prior to treatment services, such as for pregnant women and other reproductive services. This was evidenced as one of the CHWs from Sumbawanga DC said that:

‘We provide general health education and sometimes family planning, nutrition and other related health services at the household, community [through general meetings or any community gatherings], and health facility. We also provide health education during the outreach programmes with the help of HCWs who provide health education at the community level and after clients come to the health facility. We visit the villages once or twice a month to provide health education and other health services.’ (Participant #4)

### Theme 2: Challenges for adopting comprehensive health-seeking behaviour

The participants indicated some challenges for the community to adapt comprehensive HSB with the following sub-themes: frequent stockout of drugs, overburdened HFs as a result of large catchment areas, cultural and behavioural barriers to timely health-seeking, unreliable and inadequate transport systems, poor continuity of preventive health-seeking after campaign and low health insurance coverage.

#### Sub-theme 2.1: Frequent stockout of drugs in health facilities

The interviewed participants indicated that the major challenge discouraging community members from seeking healthcare services at HFs is the frequent shortage of medicines. Although patients may receive other necessary services, the lack of medicines often discourages them from returning to the facility in the future. Those who can afford to buy medicines may choose to go directly to private pharmacies instead of visiting HFs. In addition, patients accessing care through exemption schemes are even more discouraged by the persistent unavailability of medicines, as noted by one respondent from Iringa DC:

‘… for consultation fees, counselling and laboratory tests are offered free of charge for the patients with exemptions but still a challenge on the availability of medicines at the health facility … we normally give patients the available medicines, however, if there are no medicines at the health facility, those with money go to buy medicines at the private pharmacy, and those with no money go back home to look for money for buying medicines.’ (Participants #10)

#### Sub-theme 2.2: Overburdened health facilities because of large catchment areas

Participants reported that some primary HFs serve large catchment areas covering multiple villages and a high number of community members, creating a heavy workload and limiting timely access to services. This situation may reduce the ability of facilities to provide continuous and comprehensive care and may discourage community members from seeking care early. This was evidenced by one of the participants from Iringa DC who indicated that:

‘… in this dispensary, we serve three villages … and it is estimated to serve about six thousand or more people from these villages.’ (Participant #7)

#### Sub-theme 2.3: Cultural and behavioural barriers to timely health-seeking

Participants reported that cultural and behavioural factors pose significant challenges to timely health-seeking among community members. Many individuals tend to delay seeking care until their illness becomes severe, rather than visiting HFs at the early stages of sickness or for routine checkups. This tendency is influenced by certain cultural beliefs, where visiting an HF while still healthy is perceived as inviting misfortune or illness. Such misconceptions discourage preventive health practices and limit the uptake of early diagnosis and treatment services. Consequently, these cultural and behavioural barriers continue to undermine efforts to promote proactive and comprehensive HSB within the community. As reported by one of the HCWs from Sumbawanga DC:

‘This is a challenge caused by the local belief of the area, most of the people come to the health facility when they are too sick and very few come to check for their health before illness. Only a few community members come to check for HIV status, blood pressure and blood sugar levels but not for the majority of the community members for regular checkups.’ (Participant #9)

#### Sub-theme 2.4: Unreliable and inadequate transport systems

Participants reported that limited and unreliable transport options were a major barrier to accessing HFs within the study communities. They explained that many areas lacked dependable public transport services, partly because of inadequate transport infrastructure, such as poor road networks, especially in remote villages. As a result, community members often faced difficulties reaching HFs in a timely manner, particularly during emergencies or when seeking routine care. Participants noted that many clients commonly travelled long distances on foot, while others relied on bicycles or motorcycles as alternative means of transport. One participant from Sumbawanga DC explained that:

‘… most of them come on foot, some come by bicycles and motorcycles. But generally, means of transportation to health facilities is a major challenge in our areas.’ (Participant #8)

#### Sub-theme 2.5: Poor continuity of preventive health-seeking after campaigns

Participants reported that although community members often respond positively to outreach campaigns and attend screening services when they are offered free of charge, many do not sustain the behaviour by returning for routine health checkups and follow-up visits. This pattern limits the adoption of comprehensive HSB because preventive and early detection services require continuous engagement beyond one-off campaign events. One participant explained that:

‘… though we normally conduct a health campaign once a year to make people come to the facility to check their health status. … we advised people to come and check their blood sugar, blood pressure, and cervical cancer for free of charge. Most people attended during a day of the campaign but are not seen after the campaign….’ (Participant #11)

#### Sub-theme 2.6: Low health insurance coverage

Participants reported that low health insurance coverage within the community limits access to health services and reduces health-seeking behaviour. They explained that many community members, particularly in rural areas, were not enrolled in any health insurance scheme and therefore relied on out-of-pocket payments when seeking care. This financial barrier was described as a key reason for delayed or forgone use of health services, as individuals without insurance were often unable to afford consultation fees, diagnostic tests, and treatment costs. Participants further noted that only a small proportion of uninsured individuals could manage to pay for services, which contributed to low and inconsistent utilisation of formal healthcare. One of the participants commented:

‘… most of the patients in our locality are not covered by health insurance which leads to a low level of health-seeking behaviour. … those who have no health insurance, only a few community members who can afford the cost at the health facility.’ (Participant #1)

## Discussion

The findings of this study highlight both opportunities (enablers) and challenges (barriers) in adopting comprehensive primary PHC-HSB as perceived by healthcare providers from the selected districts of Tanzania. The findings showed that enablers for adopting HSBs include: existence of exemptions and waivers, presence of special clinics, benefits of early seeking behaviour, availability of outreach services, community’s interest in health issues, presence of CHWs and health education provided at the household level. Evidence indicating the existence of barriers to HSB also emerged including: frequent stockout of drugs, overburdened HFs as a result of large catchment areas, cultural and behavioural barriers to timely health-seeking, unreliable and inadequate transport systems, poor continuity of preventive health-seeking after campaigns and low health insurance coverage. The results align with and contrast with existing literature on PHC implementation in low-resource settings.

### Opportunities (enablers) for adopting comprehensive primary healthcare-seeking behaviour

Exemptions and waivers for vulnerable groups (e.g. pregnant women, under-five children, elderly and disabled individuals) are the financial interventions put in place to facilitate healthcare access, reducing financial barriers and hence protecting the vulnerable group from experiencing catastrophic health expenditure (CHE). Key informants for this study acknowledged the existence of such intervention and its role in facilitating HSB among the community members who receive services at the selected facilities in Tanzania. Possible explanation for this is that people who belong to the exemption groups do not pay cash for health services like consultation fees, laboratory tests and drugs. This attracts the community members to increase HSB for the early detection of different diseases.^20,21^ Similar findings were reported in Ghana, where fee exemptions improved maternal and child health service utilization.^22^ However, to the contrary, Chuma et al. found that exemptions and waivers are ineffective in protecting the poor and hence hinder the accessibility of health services.^23^ This could be explained by challenges such as reimbursement delays, poor quality of care received by the exempted groups and health providers not giving exemptions to those eligible for it and ending up paying out of their pockets.

The presence of special clinics for chronic diseases (e.g. diabetes, hypertension) was identified as an opportunity to enhance HSB. Interviewed HCWs suggested that such clinics can attract individuals to HFs for the management of specific conditions, which may, over time, foster a habit of regular attendance and encourage early detection of other health problems. This aligns with studies from South Africa and Uganda, where the presence of chronic disease clinics improved patient follow-up and early detection.^24,25^ However, unlike in urban settings where such clinics are well-established, rural areas in Tanzania face staffing and resource shortages, limiting their effectiveness.^26,27^

Implementation of outreach services provides various health services including vaccinations, screening and counselling for Non Communicable Diseases (NCD) and HIV patients, maternal and child health, as well as caring for elderly people. During the interview with CHWs and CHWs, outreach service was found to be an opportunity in ensuring HSBs among members in the local communities of the selected districts in Tanzania. The outreach services are normally provided to those communities residing far from the HFs and reduce the distance covered by clients to access health services. Different health systems in the world, especially in low-resource settings, have introduced different guidelines and strategies on how outreach services can be conducted. A report by World Health Organization (WHO) and United Nations Children’s Fund (UNCEF) stipulates different types of outreach services covered and how they should be conducted.^28,29^ Additionally, a report by Health Outreach Partners (HOP),^30^ highlights the importance of clinical outreach such that outreach services bring primary health care close to communities, families and individuals who may face barriers in seeking and accessing care if they visited the health centre sites. Clinical outreach reduces financial barriers and serves time that could be spent by individuals and communities to access care at the HFs. Our findings concur with other studies, which explain the importance of outreach programmes which may increase access to health services among people, especially in rural areas, hence increasing HSB among the community members and also providing key professional support to those HCWs who are posted in hard to reach areas.^28,30,31^

The presence of CHWs in the community was found to be an opportunity for community members to develop HSB. This may be because of the efforts made by CHWs in conducting follow-ups with members of all households regarding health matters in their streets or villages. This finding is similar to findings reported by Ndambo et al. which explains that CHWs can build personal resources of trust, gratitude and hope within the communities, fostering positive interpersonal relationships between themselves, communities and HF staff, which may increase HSB.^32^ Additionally, Geldsetzer et al. found that home-based CHW intervention in urban Tanzania significantly increased the proportion of women delivering at the facility.^33^ In contrast, a randomised controlled trial by Nance et al. in Tanzania assessed whether a community-level CHW intervention could improve short-term retention in care and adherence to antiretroviral therapy (ART) among pregnant women living with HIV. Although the intervention was reported to be feasible and acceptable, it did not demonstrate strong effects on most Preventions of Mother to Child Transmission (PMTCT) indicators; however, it may have contributed to improved ART adherence among postpartum women living with HIV.^34^ However, a report by WHO highlights the important roles of CHWs, which may serve as an opportunity for adopting comprehensive health-seeking behaviour. Such roles include: delivering diagnostic, treatment or clinical care, encouraging uptake of health services, providing health education and behaviour change motivation, data collection and record-keeping, improving relationships between health system functionaries and community members and providing psychological support to the community, family and individuals.^35^

Health education provided at the household level by HCWs and CHWs was seen as a facilitator for HSB. This suggests that for attaining comprehensive HSB, health education is crucial, as recommended by Mulikuza.^36^ This is similar to the findings reported by Adongo and Asaarik in the study conducted in rural settings,^37^ which emphasised the necessity of increasing investment in public consultation, household education and other related health education methods, which may play an effective role in the spread of health education to improve the HSB among community members. Furthermore, another study from Ghana, which aimed at assessing the health-seeking behaviours of rural dwellers in under- resourced communities, found a significant association between education status and HSB. They concluded that health promotion and education policies for the community should be emphasised to bring about improvement in health-seeking among rural dwellers.^38^ Similarly, a study by Mulikuza et al., found that women with formal education were more likely to seek maternal care compared to those without.^12^ The study emphasised that countries in the sub-Saharan region need to adapt best practices for promoting healthcare education in the community so as to increase healthcare utilisation for under-five children and their mothers.

Additionally, this finding indicates that many community members were interested in receiving health-related information within their localities. Participants described that when HCWs or CHWs visit the communities, they are generally met with positive cooperation and engagement on health matters. This receptiveness suggests that communities are willing and ready to participate in health education activities and other outreach services, which may facilitate the adoption of HSB through improved awareness and stronger community–provider interactions. Healthcare providers also receive support and cooperation from the community leaders who facilitate the meeting between the community and the health providers by passing on information to the community and assisting in gathering people at the meeting points. Findings from a study done in Rwanda by Uwizeye in 2022 show that regular audience surveillance assists in determining whether messages are reaching target audiences and are being accepted and adopted. In addition, the study also found that CHWs are an important means of relaying messages, especially in the hardest to reach communities.^39^

### Challenges (barriers) for adopting comprehensive health-seeking behaviour

Frequent stockouts of essential medicines at HFs emerged as a key challenge from the key informant interviews. Participants explained that medicine unavailability discouraged community members from seeking care at HFs, as they perceived visits to be unhelpful if prescribed drugs are not available. As a result, stockouts are viewed as a major deterrent to timely health-seeking, potentially contributing to delays in care and reduced trust in the health system. Similar findings were observed by Kuwawenaruwa in his study, which assessed the effects of medicine availability and stockouts on healthcare utilisation in the Dodoma region, Tanzania. He found that the household’s healthcare utilisation was positively and significantly associated with continuous availability of all essential medicines for the surveyed HFs.^40^ The findings were also consistent with studies in Kenya and Zambia^41,42^ as well as a study by Twaweza in Tanzania.^43^ Unlike in Rwanda, where supply chain improvements reduced stockouts, Tanzania’s decentralised procurement system still faces logistical challenges.^44^

Overburdened HFs as a result of large catchment areas emerged as a major barrier to adopting comprehensive HSB. The findings indicate that a limited number of HFs were serving multiple and widely dispersed villages, resulting in large catchment populations and increased pressure on available services. This imbalance between service demand and facility availability constrained timely access to care and discouraged routine and preventive health-seeking among community members. In addition to large catchment areas, physical access was further complicated by unreliable means of transport and inadequate transport infrastructure. As a result, most patients in the study districts relied on motorcycles, bicycles, or walking to reach HFs. Similar findings have been reported in rural Ghana, where Adongo et al. found that motorcycles, bicycles and walking were the most common means of transport used to access HFs.^38^ Evidence from rural Ghana and Kenya also highlights that limited transport options restrict access to care and contribute to delayed health-seeking.^45,46^ Furthermore, studies from Kenya and Nigeria have shown that distance to HFs is associated with frequency of clinic attendance, choice of HF, cost of care, and delays in receiving services.^45,47^ Therefore, taking health services closer to the community could improve HSB and address the barriers associated with distance and transportation issues.

The study revealed that delayed healthcare-seeking behaviour, influenced by cultural and behavioural barriers to timely health-seeking, poses a significant challenge to the adoption of comprehensive HSB. Healthcare providers reported that many community members do not proactively visit HFs for early checkups, often waiting until their conditions become severe before seeking medical attention. This delay is further worsened by a deeply rooted culture of not seeking care early by majority of the community members, which sometimes discourages timely healthcare utilisation or leads individuals to seek alternative treatments. These findings align with a 2021 Tanzanian study by Mulikuza et al., and Bad and Maliganya 2024 which highlighted how cultural and religious practices shape health-seeking behaviour.^12,48^

Additionally, research from sub-Saharan Africa by Latunji and Akinyemi found that traditional beliefs, particularly regarding childhood illnesses, often lead parents to prioritise traditional healers over formal healthcare services, resulting in treatment delays.^49^ These cultural barriers highlight the need for community sensitisation programmes to promote early healthcare-seeking and integrate culturally appropriate health interventions.

The study further found that limited health insurance coverage restricts healthcare access and discourages HSB among community members. Healthcare providers reported encountering many uninsured patients during facility visits and outreach sessions, with a number of households lacking insurance coverage, especially in rural areas. These findings align with multiple Tanzanian and African studies that have found a positive association between health insurance and healthcare utilisation.^50,51,52^ Health insurance serves as a financial risk protection against catastrophic health expenditures and is important for achieving Universal Health Coverage, ensuring equitable access to care without financial hardship^13,53,54,55^ However, the study revealed that many rural residents are uninsured, hence limiting their ability to afford healthcare services and reducing formal care-seeking. This suggests that expanding health insurance coverage could enhance health-seeking behaviour, as evidenced by findings from Tabora, where insured elderly individuals utilised healthcare more than their uninsured counterparts.^56^ However, this contrasts with other studies in which insurance did not significantly influence help-seeking behaviour,^17,57^ indicating contextual variations in insurance impact.

The study also identified personal risk behaviours as a key factor influencing health-seeking practices. Participants noted that individuals engaging in high-risk behaviours, such as excessive alcohol consumption or unhealthy lifestyles, were less likely to seek timely healthcare. This finding is supported by Tungu et al., who found that individuals with chronic disease risks or unhealthy behaviours exhibited different health-seeking patterns compared to their healthier counterparts.^56^ Such behaviours contribute to delayed care-seeking, as individuals often wait until symptoms become severe before visiting HFs. Addressing these risk factors through targeted behavioural interventions could improve early healthcare utilisation and overall community health outcomes.

### Limitations of the study

This qualitative study has several limitations that should be considered when interpreting the findings. Firstly, the small sample size (15 participants) and purposive sampling may limit the generalizability of the results to broader populations. Secondly, the study was conducted in only two districts (Iringa and Sumbawanga), which may not fully represent the diverse healthcare challenges and opportunities across Tanzania. Thirdly, as a qualitative study, the findings are based on participants’ subjective perspectives, which could introduce social desirability bias, particularly among HCWs reporting on service delivery challenges. Finally, the PHC facility-based design may have overlooked community-level factors influencing health-seeking behaviours among individuals who do not frequently access formal healthcare facilities. These limitations suggest the need for further mixed-methods or multi-site studies to validate and expand on these findings.

## Conclusion

The study highlights key enablers and barriers in adopting comprehensive primary healthcare-seeking behaviours and practices (PHC-HSBs) from the perspectives of healthcare providers in Tanzania. While opportunities such as existence of exemptions and waivers, presence of special clinics, benefits of early seeking behaviour, availability of outreach services, community’s interest in health issues, presence of CHWs and health education provided at the household level, and the presence of CHWs were identified. While challenges like frequent stockout of drugs, overburdened HFs as a result of large catchment areas, cultural and behavioural barriers to timely health-seeking, unreliable and inadequate transport systems, poor continuity of preventive health-seeking after campaigns and low health insurance coverage persist were identified. Addressing these gaps requires policy reforms, better supply chain management and community-based interventions to strengthen PHC and shift health-seeking behaviours, especially in rural and underserved settings. To enhance the adoption of PHC-HSB, policymakers and health stakeholders should prioritise capacity-building for HCWs, community-based health education, and increased funding for PHC facilities. Additionally, integrating traditional and modern healthcare practices while addressing socio-cultural barriers could improve health-seeking behaviours. Further research should explore strategies for scaling up these interventions across similar settings in Tanzania, and should explore the perception of community members and leaders.

## References

[1] Ministry of Health Tanzania. Primary health services development programme (PHSDP) 2007 – 2017 Tanzania. [homepage on the Internet]. Health Education. 2007 [cited n.d.], p. 107. Available from: https://core.ac.uk/download/pdf/11307294.pdf

[2] WHO-UNICEF. Alma-Ata 1978 primary health care~Report of the international conference on primary health care. Geneva. 1978, p. 63.

[3] WHO. Primary health care programme in the WHO African region from Alma-Ata to Ouagadougou and beyond: The African health transformation programme: A vision for Universal Health Coverage. Geneva. 2018, p. 1–72.

[4] MOH-Tanzania. Health sector strategic plan. Heal San Fr [homepage on the Internet]. 2021, p. 1–98. Available from: https://mitu.or.tz/wp-content/uploads/2021/07/Tanzania-Health-Sector-Strategic-Plan-V-17-06-2021-Final-signed.pdf

[5] Astana Declaration. Global conference on primary health care: From Alma-Ata towards universal health coverage and the Sustainable Development Goals. Geneva: World Health Organization; 2018.

[6] Thapa G, Jhalani M, García-Saisó S, Malata A, Roder-DeWan S, Leslie HH. High quality health systems in the SDG era: Country-specific priorities for improving quality of care. PLoS Med. 2019;16(10):10–13. 10.1371/journal.pmed.1002946 PMC6776256 31581260

[7] United Republic of Tanzania. Annual health sector performance profile 2023. Dar es Salaam. 2024.

[8] WHO. Towards universal health coverage in the WHO African region: Tracking financial protection. Geneva. 2024.

[9] Ally M, Piatti-Fünfkirchen M. Tanzania health sector public expenditure review. Dar es Salaam. 2020.

[10] Dutta A, Team S. Implications for Tanzania’S fourth health sector strategic plan, and impact analyses using [homepage on the Internet]. 2015 [cited n.d.]. Available from: http://www.healthpolicyproject.com/pubs/527_FINALTZOneHealthreport.pdf

[11] World Health Organisation. Tracking universal health coverage: 2023 global monitoring report. CC BY-NC-SA 3.0 IGO. Geneva. 2023.

[12] Mulikuza J, Buhori JA, Msoline D. Assessment of health seeking behaviours among communities in Mikindani municipality. Tengeru Community Dev J. 2021;8(1):37–51.

[13] Kagaigai A, Grepperud S. The role of risk preferences: Voluntary health insurance in rural Tanzania. Health Econ Rev. 2023;13(1):1–15. 10.1186/s13561-023-00432-z 37004684 PMC10067166

[14] Langat EC, Ward P, Gesesew H, Mwanri L. Challenges and opportunities of universal health coverage in Africa: A scoping review. Cent Excell Women Child Heal. 2025;1:20. 10.3390/ijerph22010086 PMC11764768 39857539

[15] WHO. Building health systems resilience for universal health coverage and health security during the COVID-19 pandemic and beyond. Geneva. 2021.

[16] Kassim M. A qualitative study of the maternal health information-seeking behaviour of women of reproductive age in Mpwapwa district, Tanzania. Health Info Libr J. 2021;38(3):182–193. 10.1111/hir.12329 33052617 PMC8518957

[17] Athumani KF, Mboineki JF. Healthcare facility factors associated with health-seeking behavior among secondary school students in the Dodoma region: An analytical cross-sectional study. Front Public Heal. 2025;13:1–7. 10.3389/fpubh.2025.1457318 PMC11983427 40213432

[18] Lyimo EJ, Msangi M, Zangira AJ, et al. PLOS global public health healthcare-seeking behaviours among mother’s having under-five children with severe wasting in Dodoma and Mbeya regions of Tanzania – A qualitative study. PLoS Glob Public Health. 2024;4(1):1–18. 10.1371/journal.pgph.0001943 PMC10773934 38190374

[19] Creswell JW. Qualitative inquiry and research design: Choosing among five approaches. 2nd ed. Thousand Oaks, CA: SAGE Publications, 2007; p. 414.

[20] Munishi V. Assessment of user fee system: Implementation of exemption and waiver mechanisms in Tanzania. Successes and challenges [Msc Physics]. Univeristy of Cape Town. UOFS; 2008.

[21] Mubyazi GM. The Tanzanian policy on health-care fee waivers and exemptions in practice as compared with other developing countries: Evidence from recent local studies and international literature. East Africa J Public Heal. 2004;1(1):1–10.

[22] Agyepong IA, Kwamie A, Frimpong E, et al. Spanning maternal, newborn and child health (MNCH) and health systems research boundaries: Conducive and limiting health systems factors to improving MNCH outcomes in West Africa. Heal Res Policy Syst. 2017;15(suppl 1):54. 10.1186/s12961-017-0212-x PMC5516848 28722556

[23] Chuma J, Gilson L, Molyneux C. Treatment-seeking behaviour, cost burdens and coping strategies among rural and urban households in Coastal Kenya: An equity analysis. Trop Med Int Heal. 2007;12(5):673–686. 10.1111/j.1365-3156.2007.01825.x 17445135

[24] WHO. Early detection of tuberculosis An overview of approaches, guidelines and tools. WHO Library Cataloguing-in-Publication Data. Geneva. 2011, vol. 21.

[25] Mayega RW, Ekirapa E, Kirunda B, et al. What kind of life is this?’ Diabetes related notions of wellbeing among adults in eastern Uganda and implications for mitigating future chronic disease risk. BMC Public Health. 2018;18(1):1–14. 10.1186/s12889-018-6249-0 PMC6307159 30587168

[26] Gizaw Z, Astale T, Kassie GM. What improves access to primary healthcare services in rural communities? A systematic review. BMC Prim Care. 2022;23(1):1–16. 10.1186/s12875-022-01919-0 PMC9724256 36474184

[27] Shayo EH, Murdoch J, Kiwale Z, et al. Management of chronic conditions in resource limited settings: Multi stakeholders’ perception and experiences with receiving and providing integrated HIV, diabetes and hypertension services in Tanzania. BMC Health Serv Res. 2023;23(1):1–13. 10.1186/s12913-023-10123-4 37858150 PMC10585858

[28] WHO and UNICEF. Community-based health care, including outreach and campaigns, in the context of the COVID-19 pandemic [homepage on the Internet]. WHO. 2020 [cited n.d.]. Available from: http://www.wipo.int/amc/en/mediation/rules%0Ahttps://www.who.int/publications/i/item/WHO-2019-nCoV-Comm_health_care-2020.1

[29] WHO. Outreach services as a strategy to increase access to health workers in remote and rural areas. Geneva. 2011. 26269879

[30] Lee S, Spurgeon L, Lieu D, Stoimenoff K, Wielunski A. Clinical outreach [homepage on the Internet]. Health Outreach Partners (HOP) would. 2015 [cited n.d.]. Available from: www.outreach-partners.org

[31] Kshetrimayum N, Bennadi D, Siluvai S. Outreach programme – Key to success in health care access. Pharma Innov [serial online]. 2015 [cited n.d.];(4, Part A):4–7. Available from: https://search.proquest.com/scholarly-journals/outreach-programme-key-success-health-care-access/docview/1924574562/se-2?accountid=31533

[32] Ndambo MK, Munyaneza F, Aron M, Makungwa H, Nhlema B, Connolly E. The role of community health workers in influencing social connectedness using the household model: A qualitative case study from Malawi. Glob Health Action. 2022;15(1):2090123. 10.1080/16549716.2022.2090123 35960168 PMC9377265

[33] Geldsetzer P, Mboggo E, Larson E, et al. Community health workers to improve uptake of maternal healthcare services: A cluster randomized pragmatic trial in dar es salaam, Tanzania. PLoS Med. 2019;16(3):1–27. 10.1371/journal.pmed.1002768 PMC6440613 30925181

[34] Nance N, Pendo P, Masanja J, et al. Short-term effectiveness of a community health worker intervention for HIV-infected pregnant women in Tanzania to improve treatment adherence and retention in care: A cluster-randomized trial. PLoS One. 2017;12(8):1–16. 10.1371/journal.pone.0181919 PMC5578486 28859083

[35] WHO. What do we know about community health workers? A systematic review of existing reviews. Human Resources for Health Observer Series no 19. Geneva. 2020, vol. 17.

[36] Mulikuza J. Assessment of health seeking behaviours among communities in Mikindani Municipality. Tengeru Community Dev J. 2021;8(1): 2021.

[37] Lavington SH. Problems faced by a computer science department in a developing country. Pap SIGCSE Bull. 1978;14(4):128–131. 10.1145/990555.990603

[38] Adongo WB, Asaarik MJA. Health seeking behaviors and utilization of healthcare services among rural dwellers in under-resourced communities in Ghana. Int J Caring Sci. 2018;11(2):840–850.

[39] Uwizeye G, Mwali AK, Radeny S. Health-seeking behavior through strategic social and behavior change. IntraHealth Int. 2022;1–6.

[40] Kuwawenaruwa A, Wyss K, Wiedenmayer K, Metta E, Tediosi F. The effects of medicines availability and stock-outs on household’s utilization of healthcare services in Dodoma region, Tanzania. Health Policy Plan. 2020;35(3):323–333. 10.1093/heapol/czz173 31942625 PMC7152726

[41] Toroitich AM, Dunford L, Armitage R, Tanna S. Patients access to medicines – A criticalreview of the healthcare system in Kenya. Risk Manag Healthc Policy. 2022;15:361–374. 10.2147/RMHP.S348816 35256867 PMC8898182

[42] Gallien J, Leung NHZ, Yadav P. Inventory policies for pharmaceutical distribution in Zambia: Improving availability and access equity. Prod Oper Manag. 2021;30(12):4501–4521. 10.1111/poms.13541

[43] Wales J, Tobias J, Malangalila E, Swai G, Wild L. Stock-outs of essential medicines in Tanzania: A political economy approach to analysing problems and identifying solutions. Dar es Salaam: Twaweza. 2014, p. 38.

[44] Mujinja PGM, Mackintosh M, Justin-Temu M, Wuyts M. Local production of pharmaceuticals in Africa and access to essential medicines: ‘Urban bias’ in access to imported medicines in Tanzania and its policy implications. Glob Health. 2014;10(1):1–12. 10.1186/1744-8603-10-12 PMC4021829 24612518

[45] Mwaura LW, Wandibba S, Olungah CO. Effect of distance on access to health services among women with type 2 diabetes in a rural community in Kenya. African J Diabetes Med [serial online]. 2017 [cited n.d.];25(1):18–20. Available from: http://www.africanjournalofdiabetesmedicine.com/articles/may_2017/8.AJDM-612.pdf%0Ahttp://ovidsp.ovid.com/ovidweb.cgi?T=JS&PAGE=reference&D=emed18&NEWS=N&AN=616943323

[46] Ansah EK, Gyapong M, Narh-Bana S, Bart-Plange C, Whitty CJM. Factors influencing choice of care-seeking for acute fever comparing private chemical shops with health centres and hospitals in Ghana: A study using case-control methodology. Malar J. 2016;15(1):1–9. 10.1186/s12936-016-1351-1 27225480 PMC4880965

[47] Okojie PW, Lane R. Health care options and factors influencing health seeking behavior in a rural community in Nigeria: A cross-sectional study. Christ J Glob Heal. 2020;7(2):83–92. 10.15566/cjgh.v7i2.335

[48] Bad L, Maliganya W. The legacy of Mwalimu Nyerere in leadership and socio-economic development in a new era of industrialization. In Proc Second Acad Conf Commem Late Mwalimu Jul Kambarage Nyerere, First Pres United Repub Tanzania Father Nation, held Mwalimu Nyerere Meml Acad Kivukoni Campus, Da. 2024, p. 16–32.

[49] Latunji OO, Akinyemi OO. Factors influencing health-seeking behaviour among civil servants in Ibadan, Nigeria. Ann Ibadan Postgrad Med. 2018;16(1):52–60. PMC6143883 30254559

[50] Kagaigai A, Anaeli A, Grepperud S, Mori AT. Healthcare utilization and catastrophic health expenditure in rural Tanzania: Does voluntary health insurance matter? BMC Public Health. 2023;23:1–13. 10.1186/s12889-023-16509-7 37592242 PMC10436390

[51] Kamuzora P, Gilson L. Factors influencing implementation of the community Hhalth fund in Tanzania. Health Policy Plan. 2007;22(2):95–102. 10.1093/heapol/czm001 17299023

[52] Borghi J, Maluka S, Kuwawenaruwa A, et al. Promoting universal financial protection: A case study of new management of community health insurance in Tanzania. Heal Res Policy Syst. 2013;11(1):21. 10.1186/1478-4505-11-21 PMC3686629 23763711

[53] Marwa B, Njau B, Kessy J, Mushi D. Feasibility of introducing compulsory community health fund in low resource countries: Views from the communities in Liwale district of Tanzania. BMC Health Serv Res. 2013;13(1):1–7. 10.1186/1472-6963-13-298 23924271 PMC3750583

[54] Asaye AW, Engidaw MT. Determinants of community based health insurance implementation: Evidence from South Gondar Zone, Amhara Region. J Women’s Health Care. 2020;10(4):528. 10.35248/2167-0420.21.10.528

[55] Aregbeshola BS, Khan SM. Out-of-pocket payments, catastrophic health expenditure and poverty among households in Nigeria 2010. Int J Heal Policy Manag. 2018;7(9):798–806. 10.15171/ijhpm.2018.19 PMC6186489 30316228

[56] Tungu M, Amani PJ, Hurtig AK, et al. Does health insurance contribute to improved utilization of health care services for the elderly in rural Tanzania? A cross-sectional study. Glob Health Action. 2020;13(1):1841962. 10.1080/16549716.2020.1841962 33236698 PMC7717594

[57] Kumah E, Asana Y, Agyei SK, Kokuro C, Ankomah SE, Fusheini A. Does health insurance status influence healthcare-seeking behavior in rural communities? Evidence from rural Ghana. Heal Policy OPEN. 2024;6:100119. 10.1016/j.hpopen.2024.100119 PMC11047188 38680189

